# The effects of sulphide on growth and behaviour of the thiotrophic *Zoothamnium niveum* symbiosis

**DOI:** 10.1098/rspb.2007.0631

**Published:** 2007-07-27

**Authors:** Christian Rinke, Raymond Lee, Sigrid Katz, Monika Bright

**Affiliations:** 1 Department of Marine Biology, University of ViennaAlthanstrasse 14, 1090 Vienna, Austria; 2 School of Biological Sciences, Washington State UniversityPullman, WA 99164-4236, USA

**Keywords:** symbiosis, sulphide, bacteria, protist, cultivation, flow-through

## Abstract

*Zoothamnium niveum* (Ciliophora, Oligohymenophora) is a giant, colonial marine ciliate from sulphide-rich, shallow-water habitats, obligatorily associated with the ectosymbiotic, chemoautotrophic, sulphide-oxidizing bacterium ‘*Candidatus* Thiobios zoothamnicoli’. The aims of this study were to characterize the natural habitat and investigate growth, reproduction, survival and maintenance of the symbiosis from *Corsica*, France (Mediterranean Sea) using a flow-through respirometer providing stable chemical conditions. We were able to successfully cultivate the *Z. niveum* symbiosis during its entire lifespan and document reproduction, whereby the optimum conditions were found to range from 3 to 33 μmol l^−1^ΣH_2_S in normoxic seawater. Starting with an inoculum of 13 specimens, we found up to 173 new specimens that were asexually produced after only 11 days. Observed mean lifespan of the *Z. niveum* colonies was approximately 11 days and mean colony size reached 51 branches, from which rapid host division rates of up to every 4.1 hours were calculated. Comparing the ectosymbiotic population from *Z. niveum* colonies collected from their natural habitat with those cultivated under optimal conditions, we found significant differences in the bacterial morphology and the frequency of dividing cells on distinct host parts, which is most likely caused by behaviour of the host ciliate. Applying different sulphide concentrations we revealed that the symbiosis was not able to survive without sulphide and was harmed by high sulphide conditions. To our knowledge, this study reports the first successful cultivation of a thiotrophic ectosymbiosis.

## 1. Introduction

Invertebrates and protists are prominent hosts of chemolithoautotrophic, sulphur-oxidizing (thiotrophic) bacteria. The hosts have to fulfil the needs of their symbiotic microbes for oxygen as an electron acceptor, as well as reduced sulphur species such as sulphide (thereafter used for the sum of H_2_S, HS^−^ and S^2−^) as an electron donor. The symbionts themselves can live endosymbiotically inside host cells, as in the well-studied *Riftia pachyptila* symbiosis (Stewart & Cavanaugh 2006*a*), or between host tissues (*Olavius algarvensis* symbiosis; Dubilier *et al*. 2006), or ectosymbiotically on some parts of the outer surface of eukaryotes (*Rimicaris exoculata* symbiosis; Polz & Cavanaugh 1995).

The most direct way to observe host performance as well as interactions between host and symbiont is to cultivate the symbiosis in the laboratory under conditions similar to the natural habitat. This has been demonstrated using flow-through respirometer aquaria with artificially added and continuously monitored chemical species such as sulphide and oxygen, maintaining thiotrophic endosymbioses of bivalves (Anderson *et al*. 1987; Steward & Cavanaugh 2006*b*), and vestimentiferan tubeworms (Childress *et al*. 1984; Girguis *et al*. 2002; Dattagupta *et al*. 2006; Girguis & Childress 2006). So far no thiotrophic ectosymbioses have been maintained in culture, even though a variety of such symbioses are known from marine sulphidic environments (Ott *et al*. 1998).

**Figure 1:**
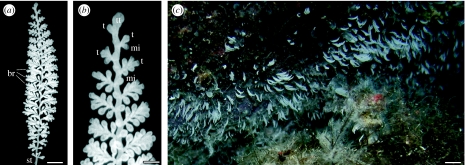
(*a*) Single *Zoothamnium niveum* colony consisting of a central stalk (st) with alternate branches (br). Scale bar, 0.5 mm. (*b*) Magnification of the tip of a *Z. niveum* colony, showing the top terminal zooid (tt), terminal zooids (t) and microzooids (mi) on branches. Scale bar, 50 μm. (*c*) *Zoothamnium niveum* colonies growing on a slightly overhanging rock wall (upper portion of picture) in a band-like distribution in close proximity to *Posidonia oceanica* and *Cystoseira* spp. debris (lower portion of picture) at observation site 3 in the Bay of Calvi, France. Scale bar, 10 mm.

*Zoothamnium niveum* has been described from mangrove areas at the Belize Barrier Reef and was also found in the Red Sea and at the coast of Florida (Bauer-Nebelsick *et al*. 1996*a*; Clamp & Williams 2006). This ciliate obligatorily lives with ectosymbiotic bacteria (Rinke *et al*. 2006). The feather-like *Z. niveum* colony exhibits a central stalk with branches occurring alternately on the stalk (figure 1) and three cell morphotypes on the branches: microzooids are the feeding stages; macrozooids are the dispersal stages, capable of leaving the colony as large swarmers to build a new colony after settlement; and terminal zooids are responsible for asexual reproduction by longitudinal fission (Bauer-Nebelsick *et al*. 1996*a*,*b*). Within the latter, two subtypes can be distinguished: terminal zooids on the branches building new zooids and the top terminal zooid present only with a single cell located on the tip of each colony (figure 1). Every division of the top terminal zooid generates a new terminal zooid and initiates the formation of a new branch. Therefore, the number of branches of a colony is equivalent to the number of divisions of the top terminal zooid and vice versa.

The symbiotic coat of *Z. niveum* consists of a bacterial monolayer, whereby two bacterial morphotypes are present. Stalk, branches, terminal- and macrozooids are covered with rod-shaped symbionts, whereas microzooids serve as substrate for coccoid rods. A series of intermediate shapes between coccoid rods on the oral side and rods on the aboral side of the microzooids was noted (Bauer-Nebelsick *et al*. 1996*b*). Phylogenetic analyses of specimens from the Belize Barrier Reef revealed a single pleomorphic ectosymbiont, which was described as ‘*Cand*. Thiobios zoothamnicoli’ and found to cluster with thiotrophic free-living and other symbiotic *Gammaproteobacteria* (Rinke *et al*. 2006).

The chemical microenvironment of the *Z. niveum* symbiosis inhabiting mangrove peat walls along tidal channels and ponds in the Caribbean Sea has been investigated in detail: by repeated contractions and expansions of the colony, the host changes its position between anoxic, sulphidic seawater leaking from the peat surface and sulphide-free, normoxic seawater above (Ott *et al*. 1998). Through expansions, sulphidic seawater is transported upward into the oxygenated zone. Immediately after expansion, microzooids start beating their cilia, to create a feeding current, transporting sulphide- and oxygen-containing seawater towards the zooids, thus creating an environment in which the chemoautotrophic, sulphide-oxidizing ectosymbiont thrives (Vopel *et al*. 2001, 2002). Furthermore, a simultaneous venting of groups of *Z. niveum* colonies with sulphide-rich seawater driven by pulsating boundary-layer currents could be demonstrated (Vopel *et al*. 2005). Measurements with microsensors showed that the swarmers of the ciliate preferentially settled and grew at peat surface in areas with sulphide values of 250–300 μmol l^−1^ and O_2_ values of approximately 20 μmol l^−1^ (Ott *et al*. 1998; Vopel *et al*. 2001).

The purpose of the present study, dealing with a recently discovered *Z. niveum* population from the Mediterranean Sea (Bay of Calvi, France), was to characterize the natural habitat and test the chemical conditions under which the symbiosis could be maintained or even cultivated in the laboratory. Although lacking the *in situ* gradient of sulphide and oxygen, we took the approach of using a flow-through respirometer (*in vivo*) with stable chemical conditions. Consequently, the thiotrophic symbionts received a continuous supply of all chemicals necessary for sulphide oxidation and carbon fixation regardless of the host's behaviour to control the gradient, i.e. contracting, expanding and beating of cilia. We performed a set of experiments in which the temperature and oxygen supply was kept constant but the sulphide concentration was varied over a wide range. Thus, we were able to gain valuable insights into the growth and reproduction of this symbiosis and its population dynamics. In addition, we tested whether or not any morphological differences occur between the ectosymbiotic population *in situ* and *in vivo*. To our knowledge, this study reports the first successful cultivation of a thiotrophic ectosymbiosis over its entire lifespan.

## 2. Material and methods

### (a) Study sites and specimen collection

**Figure 2:**
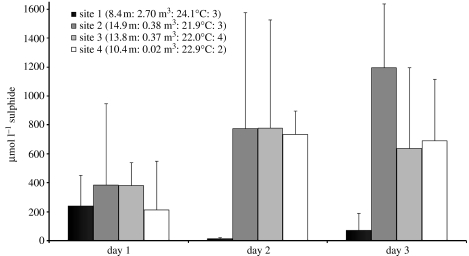
Four sites of *P. oceanica* and *Cystoseira* sp. debris accumulations in the Bay of Calvi (Corsica, France), where *Z. niveum* colonies could be observed. *In situ* measurements of sulphide were applied during 3 days of observation. Given are site number, and, in parentheses, water depth of sites, volume of debris accumulation, recorded temperature and number of sites (1–4) of debris which were protected by rocks, whereby 4 would mean that the debris is completely surrounded by rocks.

Colonies of *Z. niveum* (Hemprich & Ehrenberg 1831) were found in the bay of Calvi in Corsica, France (Mediterranean Sea) on vertical and overhanging rocks as well as on *Posidonia oceanica* leaves in wave-protected areas. Four sites, within the range of scuba diving, were found to occur at depths between 8.4 and 14.9 m, with 0.02–2.70 m^3^ accumulations of *P. oceanica* and *Cystoseira* spp. debris. These accumulations were present between 2 September and 5 October 2005 with water temperature ranging from 21.9 to 24.1°C (figure 2). All sites appeared to be well protected to varying degrees against water movement by surrounding rocks (figure 2). Water samples for sulphide measurements, taken with a 2 ml syringe introduced 10 cm into the debris were analysed by a quantitative colorimetric assay after Cline (1969). Sulphide in the debris of all four sites ranged from 12 to 1194 μmol l ^−1^ over 3 days of monitoring. The highest sulphide values were measured at the deeper sites 2–4 (figure 2).

For laboratory experiments, *P. oceanica* leaves with *Z. niveum* attached were collected from a depth of 13.8 m (site 3) in September 2005. A phylogenetic analysis of ectosymbiotic bacteria of *Z. niveum* colonies from this sampling site revealed a very high 16S rDNA gene sequence similarity (99.66%) to the Caribbean population, suggesting that the Mediterranean and the Caribbean *Z. niveum* colonies have the same bacterial species as symbiont (C. Rinke *et al*. 2007, personal observation). Immediately after sampling, the colonies were placed into flow-through respirometer aquaria, where they were maintained throughout the experiments or fixed for molecular biology and scanning electron microscopy analyses.

### (b) Flow-through system and experimental set-up

We used an automated flow-through respirometer for our cultivation experiments. Six acrylic glass aquaria (volume approx. 6 ml) with detachable glass covers were incorporated into the flow-through respirometer and the experiments ran simultaneously for a period of 13 days. The total numbers of colonies as well as the number of branches of each colony were counted each day over a period of 11 days, and lifespan of each colony was evaluated over the entire duration of the experiment. To provide physico-chemical conditions similar to those found *in situ*, 100 μm filtered seawater was pumped into a 20 l plastic cylinder and bubbled with air until nearly 100% oxygen saturation (normoxic seawater) was reached. A peristaltic pump (Minipuls 3, Gilson International, Vienna, Austria) ensured the normoxic seawater supply to the aquaria. Sulphide was introduced by a syringe pump (KD Scientific, Inc., Holliston, MA, USA) holding five 60 μl syringes with sulphide stock solutions (ranging from 7 to 0.3 mmol l^−1^ sulphide) consisting of sodium sulphide in anoxic, distilled water, mixing with the normoxic seawater before entering the aquaria.

### (c) Ectosymbiotic morphology and calculation of frequency of dividing cells

To study the ectosymbiont morphology, we used three *Z. niveum* colonies from two sets: specimens collected from the environment (hereafter referred to as *in situ*) and those cultivated at 12 μmol l^−1^ sulphide conditions for 14 days (hereafter referred to as *in vivo*).

Prior to fixation with 2% osmium tetroxide in 0.2 μm filtered seawater for 15 min, colonies were frozen in a −20°C freezer for 20 min and subsequently transferred to room temperature, thus enabling fixation during the melting process. This procedure successfully prevented contractions of colonies and made the later observations of single zooids possible. After fixation, colonies were rinsed in PBS three times for 5 min each, dehydrated in 30 and 50% ethanol for 5 min each and stored in 70% ethanol until further treatment up to a year later. For scanning electron microscopy, specimens were further dehydrated up to 100% ethanol, chemically dried with HDMS (EMS, Hartfield, PA, USA) and sputter coated. A Philips XL 20 scanning electron microscope was used to view the colonies.

For evaluation of the ectosymbiont morphology, 15 microzooids of every colony were divided into five microscopic counting frames of equal length, starting with frame 1 on the oral part of the zooids. Additionally, three counting frames (oral, middle and aboral) were established on the top terminal zooid of every colony. In each frame, 40 bacterial cells were measured clockwise starting from the centre at a 2000× magnification. In order to determine the bacterial morphology, the major axis length (length) and the minor axis length (width) of each bacterial cell were measured and the volume was calculated considering each cell as a cylinder plus two hemispheres (van Veen & Paul 1979). The cell elongation index was defined as the ratio of length to width. The frequency of dividing cells (FDC) was calculated as ((mean number of dividing cells)/(mean number of total cells))×100. Bacteria showing an invagination but not a clear intervening zone between cells were considered to be one dividing cell (Hagström *et al*. 1979).

### (d) Statistics

Using the software SPSS v. 12.0, the modified Kolmogorov–Smirnov test of normality did not reveal normal distributions for any of the data tested, including lifespan and number of branches of colonies, as well as length, width, volume, elongation index and FDC of the symbiont. Therefore, non-parametric statistics were applied. The Mann–Whitney *U*-test with a 99% CI was used to compare two datasets. Comparisons of more than two datasets were applied with the Kruskal–Wallis *H*-test with a 99% CI, followed by the Tamhane *post hoc* multiple comparison test for unequal variances with a 99% CI.

## 3. Results

### (a) Cultivation of the symbiosis

**Figure 3:**
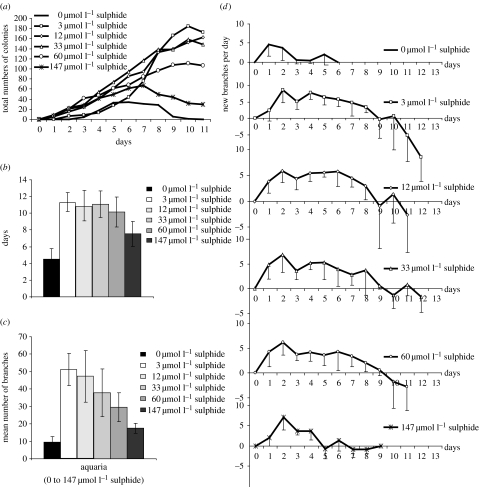
(*a*) Total numbers of *Z. niveum* colonies in the six aquaria supplied with different concentrations of sulphide counted during cultivation experiment. (*b*) Mean lifespan, (*c*) mean number of branches and (*d*) mean number of newly built branches per day of *Z. niveum* colonies in the six aquaria. Error bars in (*b*–*d*) show standard deviation.

We were able to successfully cultivate *Z. niveum* colonies during their entire life cycle and document reproduction. Starting with 13 colonies each as inoculum, all six treatments (table 1) led to asexual reproduction via swarmers from the inoculum after 24 hours. The increase of colonies, i.e. the settlement and growth of swarmers into new colonies, was strongly dependent on the amount of sulphide supplied during the 11 days of observation. In the normoxic treatment, a maximum of only 34 new colonies were observed, while in the high sulphide treatment (147 μmol l^−1^), 67 new colonies formed. We found the highest increase in the low sulphide range between 3 and 33 μmol l^−1^ with between 148 and 173 new colonies, whereas under medium sulphide conditions (60 μmol l^−1^), a maximum of 111 colonies were recorded (figure 3*a*). In addition, the rates of colony increase per day differed, ranging from minimum 15 new colonies under normoxic conditions up to maximum 57 new colonies under 3 μmol l^−1^ sulphide conditions. Furthermore, the time point when death of colonies exceeded the formation of new colonies also differed. The peak and decline in population occurred after only 5 days in the normoxic treatment, and after 7 days in the high sulphide treatment. No clear decrease was observed in the low and medium sulphide treatments until the termination of the experiment after the 11th day (figure 3*a*).

Microscopic observations further revealed that in the normoxic treatment colonies turned pale within 4 hours, whereas under the treatment with sulphide colonies remained white. Only in the high sulphide treatment, unspecific epigrowth of white filamentous bacteria was observed and the colonies showed a stunted growth.

We calculated a mean lifespan of *Z. niveum* colonies of 4.5±1.2 days (mean ±s.d.; *N*=12) under normoxic conditions, significantly shorter than under sulphidic conditions (*p*<0.000, *post hoc* Tamhane test; figure 3*b*). Under high sulphide conditions, the lifespan of 7.5±1.5 days (mean ±s.d.; *N*=12) was significantly shorter than those ranging from 10.9±1.8 (mean ±s.d.; *N*=11) to 11.4±1.1 (mean ±s.d.; *N*=11) under the low sulphide conditions between 33 and 3 μmol l^−1^ (*p*<0.000, *post hoc* Tamhane test). The longest lifespans of approximately 11 days were found under the low and medium sulphide conditions.

The size of the colonies was classified by counting the number of branches, which is a direct measure of the dividing activity of the top terminal zooid. Over the experimental time of 13 days, the mean number of branches per colony (i.e. division rate of top terminal zooid) was 9.42±3.18 (mean ±s.d.; *N*=12) under normoxic conditions and reached up to 51.27±9.29 (mean ±s.d.; *N*=13) branches under conditions with 3 μmol l^−1^ sulphide supply (figure 3*c*). Both under normoxic and high sulphide treatments, the mean number of branches was significantly lower than those under all other sulphide conditions (normoxic *p*<0.005; high sulphide *p*<0.000, *post hoc* Tamhane test). In addition, a significantly lower branch number of 29.58±8.27 (mean ±s.d.; *N*=12) under the medium sulphide supply (60 μmol l^−1^) was found compared with 51.27±9.29 (mean ±s.d.; *N*=13) branches under the 3 μmol l^−1^ sulphide conditions (*p*<0.000, *post hoc* Tamhane test; figure 3*c*).

Growth, measured as net increase of branches per day, results from the division activity of the top terminal zooid minus the loss of senescent branches. Degeneration results when loss of senescent branches equals or exceeds the division activity of the top terminal zooid. We found that the time frame of the growth phase and the degenerating phase differed between treatments. While the colonies remained in the growth phase until they died on the sixth experimental day under normoxic conditions, under high sulphide conditions, the fifth day marked the switch from growth to degeneration (figure 3*d*). Thereafter, neither growth nor a clear degeneration was found, as increase approximately equalled decrease until the ninth day during which these colonies died. In contrast, clear growth phases lasting between 9 and 10 days and clear degeneration phases up to the end of the experiments were found under all other sulphide conditions (figure 3*d*).

During the growth phase, the highest increase of new branches per day was found already on the first day of life for all *Z. niveum* colonies under normoxic conditions, but was reached on the second day for those supplied with sulphide. At this time point, the colonies had developed between 8.64±2.20 (mean ±s.d.; *N*=12; 147 μmol l^−1^ sulphide) and 11.31±1.32 (mean ±s.d.; *N*=13; 33 μmol l^−1^ sulphide) branches. After the peak of the second day, a steady increase of three to five new branches per day was found up to the eighth day. Thereafter, a decrease of new branches was observed, resulting from a decrease of division activity and/or loss of branches. Under high sulphide conditions, a decrease of new branches could already be observed after 5 days. This point was reached only after 3 days under normoxic conditions (figure 3*d*).

### (b) Ectosymbiotic morphology and FDC

Observing the symbiosis using scanning electron microscopy, we found that the specific ectosymbiotic coat was established as a consistent monolayer covering the entire host surface of *Z. niveum* colonies collected from their natural habitat (*in situ*) as well as of colonies cultivated with 12 μmol l^−1^ sulphide (*in vivo*). The evaluation of the morphology, including bacterial length, width, volume and cell elongation index of the ectosymbiotic bacteria, revealed common trends as well as significant differences within and between *in situ* and *in vivo* populations.

**Figure 4:**
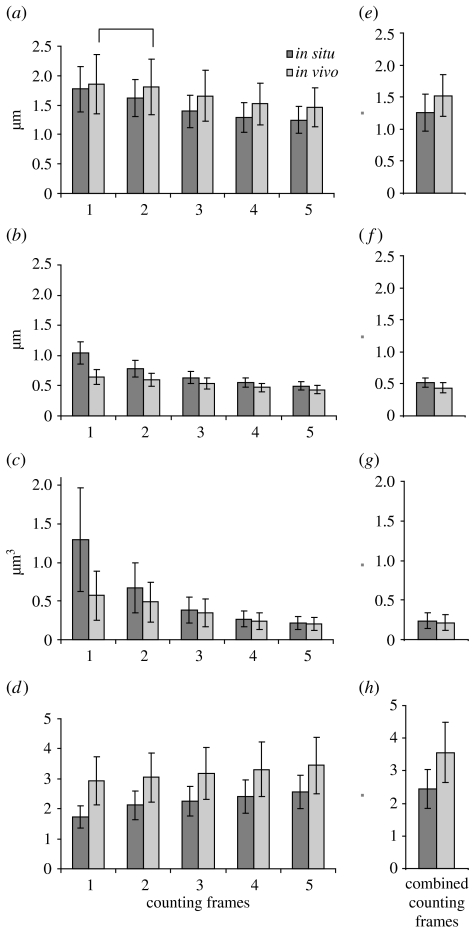
Morphology of ectosymbiotic bacteria covering the zooids of *Z. niveum,* comparing *in situ* data with *in vivo* data. Microzooids were divided into five counting frames from the oral to the aboral part (1–5) to compare (*a*) length, (*b*) width, (*c*) volume and (*d*) cell elongation index of the ectosymbiotic bacteria (*N*=1800 for each mean and s.d.). Counting frames on top terminal zooids were combined since no significant difference could be observed: (*e*) length, (*f*) width, (*g*) volume and (*h*) cell elongation index of bacteria on the top terminal zooids (*N*=360 for each mean and s.d.). All *in situ*–*in vivo* data pairs are significantly different (*p*<0.001). All microzooid counting frames differ significantly, except for bacterial length between frames 1 and 2 *in vivo* (*p*<0. 026, *post hoc* Tamhane test) which are marked with a square bracket.

On microzooids, both *in situ* and *in vivo* symbiont populations each decreased significantly in length, width and volume from the oral to the aboral parts of the microzooids, whereas the cell elongation index showed the opposite trend (figure 4 *a*–*d*). The only exception was the statistically similar symbiont lengths between frames 1 and 2 *in vivo*. On top terminal zooids, both symbiont populations showed no significant differences in length and width in all areas (i.e. counting frames); therefore, the frames were combined and the entire symbiont population was used for further analyses.

Differences between the *in situ* and the *in vivo* populations were found by comparing cell length and width, showing that the symbionts on the microzooids (all counting frames compared separately) as well as the total symbiont population of the top terminal zooid were significantly longer and thinner *in situ* than *in vivo* each (figure 4*a*,*b*,*e*,*f*). Especially in the oral area of the microzooids (frame 1), the bacterial width observed *in situ* (mean ±s.d.=1.046±0.183 μm; *N*=1800) was almost double the value of *in vivo* (mean ±s.d.=0.644±0.117 μm; *N*=1800), resulting in shorter, but much more voluminous cells *in situ* (mean ±s.d.=1.295±0.674 μm^3^; *N*=1800; figure 4*b*,*c*). In addition, the cell elongation index of bacteria on microzooids and top terminal zooids *in situ* was significantly lower than *in vivo* (*p*<0. 001, *post hoc* Tamhane test). Since cells with a cell elongation index around 1 are considered to be cocci and an elongation index over 1.5 points to rod-shaped cells (Sunamura *et al*. 2004), we translate the bacterial morphotype found *in situ* in the oral area of microzooids (frame 1) with a value of 1.718±0.374 (mean ±s.d.; *N*=1800) as coccoid rods. All other morphotypes reach cell elongation indexes over 2, up to a maximum of 3.444±0.935 (mean ±s.d.; *N*=1800) in the aboral area of the microzooids (frame 5) *in vivo* (figure 4*d*) and were termed rods.

Comparing the symbiont morphology of bacterial cells on top terminal zooids with those on microzooids, the top terminal zooid bacteria are similar in length, width, volume and cell elongation index to the bacteria on the aboral part (frame 4 and 5) of the microzooids (figure 4).

**Figure 5:**
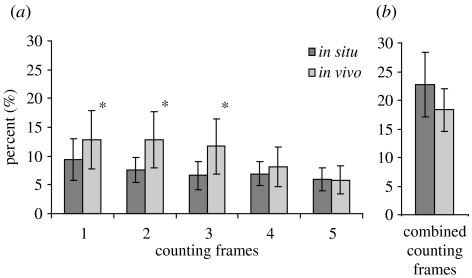
Frequency of dividing cells (FDC) of the *Z. niveum* ectosymbiont on (*a*) microzooids and (*b*) top terminal zooids *in situ* and *in vivo*. Microzooids were divided into five microscopic counting frames (1–5) of equal length, starting with frame 1 on the oral part of the zooids. Only FDC in counting frames 1–3 on microzooids differs significantly between *in situ* and *in vivo* (marked with asterisks).

The frequency of dividing bacterial cells (FDC) was highest on the oral part of the microzooids and decreased towards the aboral part, with significant differences between frames 1 and 5 (*p*<0.000, *post hoc* Tamhane test) in both populations (figure 5). FDC on the oral area of the microzooid was significantly lower *in situ* than *in vivo*, whereas no significant difference could be observed for the aboral area (figure 5). In the symbiont population from the top terminal zooid, FDC was significantly higher than from the microzooids, but no differences in activity were found between *in situ* and *in vivo* populations.

## 4. Discussion and conclusion

The cultivation of *Z. niveum* symbiosis provides us with an optimal model system to study the performance of a chemoautotrophic, sulphide-oxidizing symbiont and its host by manipulating abiotic factors in a controlled flow-through system. By investigating the host's life cycle and the population dynamics of this symbiosis *in vivo*, we also gained insights into the interactions between host and ectosymbionts. In contrast to animals that host a slow-growing thiotrophic ecto- or endosymbiont and reproduce late in life compared with protists, the model system we chose comprises a fast-growing host ciliate with a short life and reproductive cycle. Moreover, this symbiosis responded rapidly to different physical–chemical conditions allowing their performance to be manipulated easily and studied in detail.

Maintenance of endosymbiosis of siboglinid tubeworms and bathymodiolid mussels in high-pressure flow-through systems provided valuable insights into these thiotrophic symbioses (Childress *et al*. 1984; Girguis *et al*. 2002; Company *et al*. 2006). However, none of these symbioses from deep-sea hydrothermal vents have been cultivated over the entire lifespan. The same holds true for shallow-water symbiosis although nematode hosts from the subfamily *Stilbonematinae* could be kept alive several days during migration experiments (Ott *et al*. 1991), and bivalves of the families *Lucinidae*, *Solemyidae* and *Thyasiridae* were cultivated for months under laboratory conditions (Gros *et al*. 1996; Krueger *et al*. 1996; Dando *et al*. 2004). Furthermore, in contrast to endosymbioses, our model system is an ectosymbiosis in which the symbionts thrive on the host's surface directly exposed to and supplied with necessary substrates from the surrounding seawater. Thus, the *Z. niveum* ectosymbiosis enables us to directly manipulate the symbionts.

Although in nature, *Z. niveum* lives at the interface between a source of sulphide either from mangrove peat walls or from accumulations of decaying seagrass or macroalgae and overlying oxygenated seawater, for cultivation we chose a system without a gradient of oxygen and sulphide. Observing the *in vivo* performance of the *Z. niveum* symbiosis, we were not only able to cultivate the symbiosis but could also study its performance and determine the optimal sulphide conditions for cultivation. Under such constant oxygen conditions with a temperature between 24 and 25°C mimicking those found in early summer in the Mediterranean Sea, we observed that low sulphide between 3 and 33 μmol l^−1^ is optimal for *in vivo* cultivation. Total number of settled colonies, mean lifespan and colony size showed the highest values under these conditions. Based on the *in vivo* data, we propose a model for the life cycle of the *Z. niveum* symbiosis. The difference between optimal laboratory and *in situ* conditions indicates that the host behaviour has a strong impact on the symbiont morphology in the natural habitat by manipulating sulphide and oxygen availability. We also found that the symbiosis requires sulphide for survival and can tolerate permanent sulphide conditions of up to 150 μmol l^−1^.

### (a) Optimal cultivation conditions

The optimal sulphide conditions for cultivation range from 3 to 33 μmol l^−1^ sulphide, coinciding with the highest lifespan, colony size and settled colonies. Apparently, under such steady conditions, the symbionts used the reduced sulphur source in order to fix carbon continuously as they were found to maintain an uninterrupted monolayer on the host's surface and remained brilliant white. It has been shown earlier that without an external sulphide source the symbionts use their internal source, turn pale and the oxygen uptake rates drop to one-third within 4 hours indicating cessation of carbon fixation at this time point (Ott *et al*. 1998).

**Figure 6:**
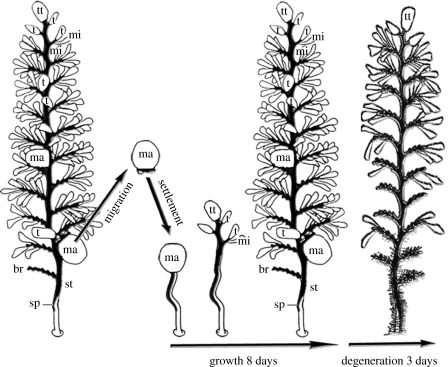
Life cycle of the peritrich colonial ciliate, *Z. niveum*. A macrozooid leaves the colony as swarmer and enters migration phase, until settlement. The young colony grows rapidly during the first 8 days, followed by a degeneration phase of 3 days. The entire colony consists of a central stalk (st) with alternate branches (br) bearing cells: macrozooids (ma); microzooids (mi); terminal zooids (t); and top terminal zooids (tt).

Based on the *in vivo* cultivation results, we propose the following model for the life cycle of the *Z. niveum* symbiosis (figure 6). Starting with a macrozooid leaving the colony as swarmer (Bauer-Nebelsick *et al*. 1996*a*, C. Rinke *et al*. 2005, personal observation), which finds a place to settle approximately within 24 hours (Ott & Bright 2004), this swarmer initiates divisions as top terminal zooid. The young colony enters a growth phase with one division every 4.1–8.2 hours until the eighth day. Thereafter, the colony reaches a degeneration phase and the net rate of branch increase drops below 0, indicating that more branches are dying than new ones are produced, until the colony dies after 11 days. The ectosymbiotic monolayer remains intact during the entire life cycle of the host, whereby the motile swarmer is covered with the ectosymbiont when leaving the *Z. niveum* colony, and thus most likely transmits the symbiont to each new host generation (Bauer-Nebelsick *et al*. 1996*a*). Newly built host surface is apparently immediately covered by the symbiont population since we never observed bacteria-free host surface under the scanning electron microscope.

On the population scale, the number of *Z. niveum* specimens increased over 14-fold during the 11 days of the experiment under optimal sulphide conditions. Thus, the original inoculum of 13 specimens doubled within the first 48 hours, making the *Z. niveum* symbiosis the fastest reproducing thiotrophic symbiosis observed so far. However, we do not know whether the release of the motile swarmers is performed during the growth phase or omitted until the colony enters the degeneration phase.

The time scale of *in situ* performance of the symbiosis remains to be studied. So far, *in situ* observations are only available for the Caribbean *Z. niveum* population living at higher temperatures than the Mediterranean population. Average water temperature in the Caribbean habitat was approximately 28.1±1.7°C during the year 2001 (Vopel *et al*. 2005), whereas the temperature in the bay of Calvi never exceeded 25°C during 2005 (personal observation). In the tropics, the colonies reach their maximum size within 4 days and have a mean lifespan of 7 days (Ott & Bright 2004; Ott *et al*. 2004). Since the metabolic rate of invertebrates and protists is strongly affected by temperature (Gillooly *et al*. 2001), most likely the symbiosis grows slower and lives longer in the more temperate waters of the Mediterranean Sea, reflecting the model proposed for *in vivo* colonies.

### (b) Natural habitat

Analysing the pore water of debris accumulations, of the newly discovered *Z. niveum* habitat in the Bay of Calvi (Mediterranean Sea), for sulphide, we observed high values of up to 1194 μmol l^−1^ and could show that the debris accumulations are a suitable habitat to sustain bacterial chemoautotrophy. However, the *Z. niveum* colonies were not observed inside the debris, but rather on vertical and overhanging rocks as well as on *P. oceanica* leaves and *Cystoseira* spp. thalli extending above the debris, placing the *Z. niveum* colonies slightly above the sulphide source. Based on large number of colonies growing and the brilliant white colour of the ectosymbiont, which is supposed to be due to elemental sulphur inclusions (Bauer-Nebelsick *et al*. 1996*a*,*b*), it is most likely that a suitable amount of sulphide reaches the *Z. niveum* colonies. Thereby we hypothesize that a combination of different active and passive transport mechanisms is present and responsible in two principal ways for sulphide support: (i) Sulphidic seawater diffuses from the debris to elevated places along vertical and overhanging areas, such as rocks, leaves or thalli in a thin layer. *Z. niveum* colonies change by contractions between this sulphidic boundary layer and the normoxic seawater, as reported for the Caribbean population (Ott *et al*. 1998). Thereby, each rapid contraction is followed by a slower expansion transporting sulphidic seawater upwards which envelopes the entire colony (Vopel *et al*. 2002). (ii) We observed long waves in the bay of Calvi at least two to three times a week during our survey in autumn 2005. These waves reached the *Z. niveum* habitats resulting in a pulsating movement of the debris accumulations (own observation), which also apparently supports the symbiosis with sulphide. Microelectrode *in situ* measurements show a similar pulsating boundary layer that alternately draws sulphide-rich and oxygenated seawater over the *Z. niveum* colonies in the Caribbean Sea (Vopel *et al*. 2005). In both (i) and (ii), the ciliary feeding current of the microzooids plays a critical role. Microzooids were reported to beat their ciliature immediately after re-expansion of the colony (Vopel *et al*. 2002) and in periods of reduced flow during flow reversals of pulsating boundary layers (Vopel *et al*. 2005). This leads to a rapid mixing of sulphide with the surrounding oxygenated seawater (Vopel *et al*. 2001).

### (c) Host impact on the symbiont morphology

**Figure 7:**
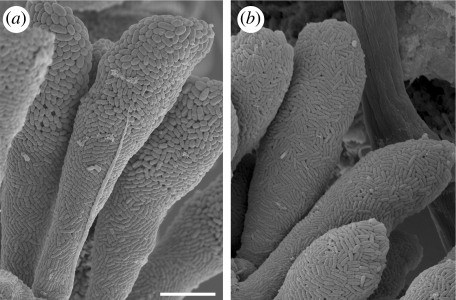
Scanning electron microscopy pictures of the *Z. niveum* symbiosis, showing ectosymbiotic bacteria. (*a*) *In situ* conditions with coccoid rods on the oral side (upper part of picture) transforming into rods on the aboral side (lower part of picture) of microzooid host cells. (*b*) *In vivo* conditions in the aquaria of the flow-through respirometer system; all parts of the microzooids are covered by the rod-shaped morphotype of the symbiont. Scale bar, 10 μm.

By replacing *in situ* gradient conditions with a constant sulphide and oxygen supply *in vivo* and concomitantly eliminating the influence of the host's behaviour (contracting, expanding and most notably beating of cilia), we demonstrate indirectly that host behaviour determines cell shape and size of the bacterial symbiont in the natural habitat. Symbiont cells on *Z. niveum* colonies from the *in situ* collection showed a transition from coccoid rods on the oral side of microzooids to rods on the aboral side, which is consistent with previous studies (Bauer-Nebelsick *et al*. 1996*a*; figure 7*a*). The polymorphic appearance of bacteria is known to be nutrition related in some free-living bacteria (White 2000), which means, in the case of the thiotrophic symbiont ‘*Cand*. Thiobios zoothamnicoli’, mainly oxygen as an electron acceptor from the overlying seawater and sulphide as an electron donor from the boundary layer (Rinke *et al*. 2006) for sulphide oxidation and generation of energy for carbon fixation. We propose that the activity of the cilia on the microzooids is responsible for the differences between the symbiont chemical environment on the oral side of the microzooids and other regions of the colony. While all symbionts gain access to similar sulphide amounts by host movement into the boundary layer and wave movements, oxygen supply for the symbionts is spatially different and manipulated actively by the host. As soon as the host emerges from the sulphidic layer, the cilia of the microzooids start beating and, thus, rapidly mix sulphide with the overlain oxygenated seawater. Thus, on the oral side of the microzooids, the symbionts have access to oxygen faster than those located on the other regions of the colony. Symbionts on the aboral part of the microzooids, as well as on stalk and branches, presumably rely on the removal of sulphidic seawater by the much slower process of chemical oxidation of sulphide (Chen & Morris 1972) to regain access to oxygen. This is also most likely true for the terminal zooids and the macrozooids, since their oral ciliature is reduced and less effective (Bauer-Nebelsick *et al*. 1996*a*,*b*). Thus, the more voluminous coccid rods on the oral area of the microzooids thrive in favourable host-regulated sulphide and oxygen conditions, enabling them to grow larger and divide faster. Whereas on host cells without the impact of a strong ciliature-created feeding current, as on the top terminal zooids, no significant increase in ectosymbiotic cell length nor width towards the oral side was observed (figure 4).

In order to test this hypothesis, we replaced these fluctuating *in situ* conditions with an equal permanent supply of sulphide and oxygen, which resulted in the ectosymbiotic bacteria on microzooids exhibiting only rod-shaped cells rather being similar to those from all other regions of the colony. This indicates that the different chemical conditions generated by the host's behaviour *in situ* were equalized, and that all ectosymbiotic cells were thriving under similar chemical conditions, even if the host still exhibited contraction and expansion behaviour. Nevertheless, a slight decrease of bacterial cell length, width and volume from the oral to the aboral part of microzooids could still be observed *in vivo*, indicating that a minor gradient of oxygen and sulphide species might still have been present along microzooid host cells.

### (d) Sulphide requirements and tolerance

All thiotrophic symbioses that use sulphide for microbial metabolism must also avoid the toxic effects of sulphide. Using our flow-through system, we tested the impact of a range of stable sulphide conditions from 0 to 147 μmol l^−1^ on the *Z. niveum* symbiosis. Cultivation under normoxic conditions revealed that the thiotrophic symbionts are not able to survive without sulphide supply for a longer time period and directly impact the hosts' growth and population increase. *Z. niveum* colonies reached less than half of their lifespan and only one-tenth of their maximum size, compared with low sulphide conditions. The ectosymbiotic bacteria turned pale within 4 hours, and the entire *in vivo* population vanished after 10 days. Net colony population decline was observed after only 5 days.

The observation that all colonies turned translucent indicates that the ectosymbiont exhausted its elemental sulphur storage under normoxic conditions. Without external sulphide supply nor internal stored sulphur, an obligate sulphide-oxidizing bacterium cannot persist. Experiments with ^14^C bicarbonate incubations demonstrated that *Z. niveum* not only uses its symbiont for nutrition, but presumably also consumes free-living bacteria from the surrounding water (Rinke 2002). Free-living bacteria were not removed in the laboratory experiments. Nevertheless, the settled *Z. niveum* colonies stayed small, survived no longer than 5 days and did not manage to sustain an *in vivo* population. So we conclude that the nutritional importance of free-living bacteria is minor. This is further supported by the fact that in nature the ciliate, *Z. niveum,* is obligatorily associated with its ectosymbiont (Bauer-Nebelsick *et al*. 1996*a*,*b*).

The minimum sulphide requirements of the symbiosis under steady *in vivo* conditions were found to be approximately 3 μmol l^−1^ or even below. However, in a flow-through respirometer system, low sulphide concentrations can be accompanied by a high rate of sulphide consumption, since depleted sulphide is replaced permanently. Nevertheless, we conclude that the ectosymbiont of *Z. niveum* is able to perform a sufficient sulphide uptake, thus enabling the thriving of the entire symbiosis, even at sulphide conditions as low as 3 μmol l^−1^ sulphide.

Only cultivation under high sulphide conditions (147 μmol l^−1^) was deleterious. The colonies had a shorter lifespan and smaller size and entered a degeneration phase after 5 days. The onset of population decrease occurred after 7 days. Preliminary cultivation experiments with a permanent sulphide supply over 200 μmol l^−1^ led to death of *Z. niveum* colonies within 3 days (data not shown). Under the applied high sulphide conditions in this study (147 μmol l^−1^), we also observed stunted growth of the *Z. niveum* colonies, including specimens with unnatural bends in the normally straight stalks and specimens, which were incapable of full expansion.

Since sulphide is a lethal highly reactive toxin known to inhibit cytochrome *c* oxidase (COX; EC 1.9.3.1) reversibly, i.e. the eukaryotic terminal enzyme of the mitochondrial electron transport chain (Dorman *et al*. 2002) in the majority of eukaryotic organisms, a steady supply of sulphide between 150 and 200 μmol l^−1^ may harm the ciliate host. However, transient exposure to higher sulphide may be tolerated. *In situ* sulphide values of up to 1194 μmol l^−1^ as measured in debris pore water probably result in pulses of sulphide to *Z. niveum* colonies that are not lethal. Furthermore, we observed that *in vivo* white filamentous bacteria, possibly thiotrophic, slowly overgrew the *Z. niveum* colonies as a biofilm encapsulating the symbiosis. This may contribute to the short lifespan and the degeneration of the population under high sulphide *in vivo* conditions.

### (e) Frequency of dividing cells

The highest FDC was observed for the ectosymbiotic bacteria on the top terminal zooids, followed by those on the oral part of the microzooids, whereas the lowest FDC was recorded for ectosymbionts on the aboral part of microzooids (figure 5). This pattern is most likely due to the different availability of free host surface. Top terminal zooids divide up to every 4.1 hours and the ectosymbionts have to maintain the bacterial monolayer covering the host, thus explaining the high FDC. On the non-dividing microzooids, new host surface is only available if ectosymbionts detach, which happens when high shear stress during stalk contraction, cell shrinkage, bunching of the zooids and beating of the cilia presumably lead to a detachment of ectosymbiotic bacteria and most likely to their consumption by the host (Vopel *et al*. 2002). Therefore, the FDC of ectosymbionts is significantly lower on microzooids than on top terminal zooids. Furthermore, the impact of the microzooids ciliature is stronger on the oral part than on the farther aboral part of the microzooid, which presumably explains why the FDC of ectosymbionts growing on the aboral part was the lowest recorded.

### (f) Conclusion

Cultivation of a thiotrophic symbiosis over its entire lifespan including reproduction leading to multiplying populations was achieved. This is a very substantial step in thiotrophic symbiosis research, since we report in the present work an easy to access, fast reproducing and cultivable model system enabling to study the symbiosis over generations under *in vivo* conditions and, therefore, to gain insights into the dynamics and interactions between host and ectosymbiont. For future approaches, an upscaling of the flow-through respirometer capacities to gain a higher biomass output for post-analyses like genomics and proteomics is desirable. Moreover, cultivation of an intact thiotrophic symbiosis provides the only ability to observe the microbial partner *in vivo,* since no chemoautotrophic symbiont has ever been cultured in a laboratory (Fisher & Girguis 2007). *In vivo* cultivation approaches will serve as basis to shed new light on the fascinating functioning of thiotrophic symbioses.

## Figures and Tables

**Table 1:** Supply of sulphide (the sum of H_2_S, HS^−^ and S^2−^) and oxygen in the six aquaria during the *Z. niveum* cultivation experiment. (Sulphide conditions were classified as high, medium and low, and conditions without sulphide supply as normoxic. Oxygen conditions in sulphide-supplied aquaria remained close to saturation level established under normoxic conditions.)

aquarium number	sulphide conditions	sulphide (μmol l^−1^)		oxygen (μmol l^−1^)	
	
		mean	s.d.	mean	s.d.
1	high	146.67	6.12	198.11	0.62
2	medium	59.57	2.53	199.79	1.21
3	low	33.22	1.83	201.58	0.82
4	low	11.89	0.30	201.85	0.72
5	low	2.65	0.77	202.53	0.45
6	normoxic	0.00	0.00	203.77	0.75

## Supplementary Material

Supplementary materials and methods Flow-through system and experimental set-up further explanation
