# Supplementary material for: The effects of sulphide on growth and behaviour of the thiotrophic Zoothamnium niveum symbiosis
Source: Proc Biol Sci. 2007 Jul 27;274(1623):2259–69. doi: 10.1098/rspb.2007.0631 (PMC1950315; doi:10.1098/rspb.2007.0631)
Supplement: Supplementary file 1 [file rspb20070631s03.pdf]

Flow-through system and experimental set-up

Oxygen, sulphide, pH and temperature were measured in the aquaria during experiments every 100 min. Oxygen was determined by an optical oxygen sensor, which was integrated in a flow-through cell and connected to the oxygen meter FIBOX (Precision Sensing GmbH, Regensburg, Germany) using polymer optical fibres. Sulphide concentrations were analysed from discrete samples using a quantitative colorimetric assay (Cline 1969) and a spectrophotometer (Dr Bruno Lange GmbH, Obergrafendorf, Germany). A pH/conductivity electrode (WTW, Weilheim, Germany) was used to check the pH value and salinity. Temperature was recorded by a Pt1000 temperature sensor connected to the oxygen meter FIBOX. A 10 port valve (VICI AG International, Schenkon, Switzerland) controlled by the software LabView v. 7.0 (National Instruments, Austin, TX, USA) was used to switch between the six aquaria, connecting one aquarium at a time to the analytical system. Five aquaria were supplied with different concentrations of sulphide (3–147 µmol l^{-1} sulphide) and oxygen (198–204 µmol l^{-1} oxygen corresponding to 96.2–98.3% oxygen saturation), and one served as normoxic seawater control (table 1). Salinity was kept at 40 psu, pH at 8.2, temperature between 24 and 25°C, and the flow rate of the seawater supply was adjusted to 100 ml h^{-1}. For each aquarium, 13 *Z.* niveum colonies of brilliant white colour bearing between 20 and 50 branches attached to a square leaf of P. oceanica (approx. 1×1 cm) served as inoculum. The settlement and growth of new *Z.* niveum colonies were observed using a dissection microscope. Each colony was numbered, branches were counted, and the location of settlement was documented once a day by drawing a 10 : 1 sketch of each aquarium, based on a 5 mm grid laid on the aquaria glass cover. For all further analyses, only newly settled colonies on chamber walls and the glass cover were considered, thus the inoculum was not included in the data analyses.
